# Women’s experiences of disrespect and abuse in Swiss facilities during the COVID-19 pandemic: a qualitative analysis of an open-ended question in the IMAgiNE EURO study

**DOI:** 10.1186/s12884-024-06598-6

**Published:** 2024-05-31

**Authors:** Alessia Abderhalden-Zellweger, Claire de Labrusse, Michael Gemperle, Susanne Grylka-Baeschlin, Anouck Pfund, Antonia N. Mueller, Ilaria Mariani, Emanuelle Pessa Valente, Marzia Lazzerini

**Affiliations:** 1 School of Health Sciences (HESAV), HES-SO University of Applied Sciences and Arts Western Switzerland, Avenue de Beaumont 21, 1011 Lausanne, Switzerland; 2 Research Institute of Midwifery and Reproductive Health, ZHAW Zurich University of Applied Sciences, Winterthur, Switzerland; 3 WHO Collaborating Center for Maternal and Child Health, Institute for Maternaland , Child Health IRCCS “Burlo Garofolo”, Trieste, Italy

**Keywords:** Disrespect and abuse in maternity care, COVID-19 pandemic, Maternal health services, Quality of care, Perinatal care, Switzerland

## Abstract

**Background:**

The COVID-19 pandemic has challenged the provision of maternal care. The IMAgiNE EURO study investigates the Quality of Maternal and Newborn Care during the pandemic in over 20 countries, including Switzerland.

**Aim:**

This study aims to understand women’s experiences of disrespect and abuse in Swiss health facilities during the COVID-19 pandemic.

**Methods:**

Data were collected via an anonymous online survey on REDCap®. Women who gave birth between March 2020 and March 2022 and answered an open-ended question in the IMAgiNE EURO questionnaire were included in the study. A qualitative thematic analysis of the women’s comments was conducted using the International Confederation of Midwives’ RESPECT toolkit as a framework for analysis.

**Findings:**

The data source for this study consisted of 199 comments provided by women in response to the open-ended question in the IMAgiNE EURO questionnaire. Analysis of these comments revealed clear patterns of disrespect and abuse in health facilities during the COVID-19 pandemic. These patterns include non-consensual care, with disregard for women’s choices and birth preferences; undignified care, characterised by disrespectful attitudes and a lack of empathy from healthcare professionals; and feelings of abandonment and neglect, including denial of companionship during childbirth and separation from newborns. Insufficient organisational and human resources in health facilities were identified as contributing factors to disrespectful care. Empathic relationships with healthcare professionals were reported to be the cornerstone of positive experiences.

**Discussion:**

Swiss healthcare facilities showed shortcomings related to disrespect and abuse in maternal care. The pandemic context may have brought new challenges that compromised certain aspects of respectful care. The COVID-19 crisis also acted as a magnifying glass, potentially revealing and exacerbating pre-existing gaps and structural weaknesses within the healthcare system, including understaffing.

**Conclusions:**

These findings should guide advocacy efforts, urging policy makers and health facilities to allocate adequate resources to ensure respectful and high-quality maternal care during pandemics and beyond.

**Supplementary Information:**

The online version contains supplementary material available at 10.1186/s12884-024-06598-6.

## Background

The consequences of the COVID-19 pandemic have been unpredictable and devastating for individuals, families, societies, and economic systems around the world [1]. Healthcare systems, including maternity care services, have been severely affected by the pandemic, as clinical guidelines and safety procedures have had to be rapidly revised and updated to contain the spread of the virus [2]. Approximately 85 000 births are observed each year in Switzerland, with the vast majority (98.3%) taking place in hospitals [3]. The rate of caesarean sections is about 32.2% [3] (17.8% elective [4]), which is higher than the European average of 25.7% [5]. Before the pandemic, Switzerland had a fertility rate of 1.48 births per woman, slightly below the EU average of 1.53 [6]. During the pandemic, fertility declined by 14.1% in European countries (5.4% in Switzerland) [7].

Because of the medical emergency caused by the pandemic, some essential aspects of maternal and newborn care have been deprioritised in health facilities [8]. Studies in various national settings have shown that during the early stages of the pandemic, women’s companions were not allowed to remain with them during labour to limit potential sources of infection. Additionally, obstetric interventions, such as caesarean sections, were performed without clear clinical indication, and women and their newborns were separated after birth if the mother tested positive for SARS-CoV-2 [9–11]. In Switzerland, specific measures and national recommendations targeting maternity services were issued, including restricting hospital visits, and limiting the presence of partners during and after childbirth [12, 13]. While these measures were implemented to control COVID-19 infection, some of them do not align with the recommendations of quality and respectful maternal care [14–16]. For instance, excluding partners and separating newborns from their mothers are particularly unrecommended practices according to the rights-based approach to maternity care [8, 17, 18]. These actions are deemed as deviations from established practice without supporting evidence [8]. Violations of respectful maternity care and disrespectful and abusive practices were observed during COVID-19, both directly and indirectly related to the pandemic context.

In 2010, seven categories of disrespect and abuse in maternity care were defined by Bowser et al. [19]. These categories include: physical abuse; non-consented care; non-confidential care; non-dignified care (including verbal abuse); discrimination based on specific attributes; abandonment or denial of care; and detention in facilities. These categories were documented by the White Ribbons Alliance [20], which sought to broaden the scope of ‘safe motherhood’ by acknowledging the importance of the relationship between caregivers and women. Building on this, the International Confederation of Midwives (ICM) subsequently used the same categories to develop the RESPECT Toolkit [21], to promote respectful maternity care for all women around the time of childbirth.

**Fig. 1:**
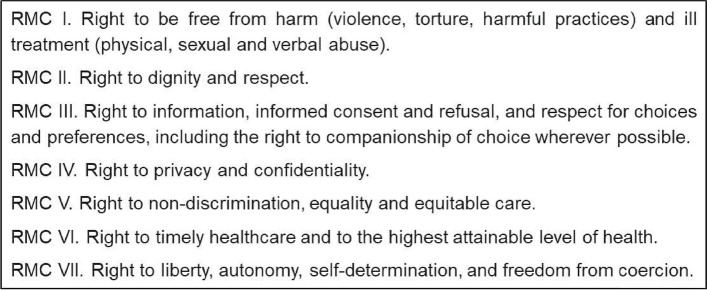
Categories of respectful care during childbirth identified by Jolivet et al. [22]). *Legend*: Abbreviation: RMC = Respectful Maternity Care

Although the focus of this paper is on disrespect and abuse in maternity care, it is important to note the corresponding rights that are designed to counteract the manifestations of these mistreatments, as outlined by Bowser et al. [19]. Figure 1 shows the categories of respectful maternity care identified by Jolivet et al. [22] in their systematic scoping review. These categories were identified by comparing two frameworks: the Respectful Maternity Care Charter developed by the White Ribbon Alliance (2011, updated 2019) [20] and the International Human Rights and the Mistreatment of Women during Childbirth by Khosla et al. [23].

Respectful maternity care is not only a fundamental human right, but it also plays a crucial role in shaping the well-being of both mothers and newborns. Negative experiences around the time of childbirth, such as disrespect or neglect of women’s wishes, discrimination, or verbal or physical abuse, can lead to poorer physical and mental health outcomes for mothers and their newborns [24]. Renfrew et al. [25] stressed the importance for maternity and neonatal services to provide pregnant women and new mothers with quality care, even in the face of unforeseen events.

Since 2020, the IMAgiNE EURO study, based on the WHO Standards for improving Quality of Maternal and Newborn Care (QMNC) [26], has been documenting QMNC during the COVID-19 pandemic in the European Region. Several studies resulting from this international project reported limitations in the QMNC provided during the COVID-19 pandemic [27–30]. Quantitative data from the IMAgiNE EURO study among 1′175 women who gave birth in Switzerland from March 2020 to February 2022, indicate that about 28% of women reported limitations in the QMNC during the pandemic [27]. However, little is known qualitatively about women’s experiences of care around the time of childbirth, and their experiences of disrespect and abuse during the COVID-19 pandemic.

This study aims to provide valuable insights into the experiences of women who gave birth in Swiss facilities during the COVID-19 pandemic, using the categories of disrespect and abuse defined in the ICM RESPECT Toolkit [21] as a framework for analysis. The results of this study will help improve maternity care provision and advocate for respectful maternal and newborn care in general, including during health crises [25].

## Methods

### Study design

This study reports qualitative data collected through the IMAgiNE EURO study “Improving Maternal Newborn Care in the European Region”. Led by the WHO Collaborating Center for Maternal and Child Health, IRCCS Burlo Garofolo, Trieste, Italy [31] and based on the WHO Standards [26], the IMAgiNE EURO study documents QMNC during the COVID-19 pandemic in more than 20 European countries, including Switzerland (ClinicalTrials.gov NCT04847336).

The Standards for Reporting Qualitative Research (SRQR) [32] were used to report this study (see Supplementary Information 1).

### Data collection

Data were collected using an online survey hosted in REDCap® as part of the IMAgiNE EURO study. The validated questionnaire was developed based on the 40 key Quality Measures of the WHO Standards for improving QMNC in health facilities [33]. Women who had given birth in Switzerland were recruited using multiple strategies, including targeted outreach to specific groups of mothers on social media, and distribution of flyers through hospitals and by independent midwives. Participants accessed the online survey via a link or QR code and could choose their preferred language from 24 available.

The data collection period covers the first 2 years of the IMAgiNE EURO study. It is important to note that, on 1 April 2022 the emergency measures related to the COVID-19 pandemic were lifted in Switzerland [34]. Women aged 18 years and older, who gave birth in Swiss’ hospitals and clinics between 1 March 2020 and 14 March 2022 and gave their consent to participate were eligible for this study. At the end of the questionnaire, a non-mandatory open-ended question allows women to provide comments for improve the QMNC: *“Do you have any suggestions to improve the quality of care at the facility where you gave birth or to improve the questionnaire?”.* However, many respondents did not provide specific suggestions, but rather gave general comments about their experience of care at the hospital where they gave birth. Although the question does not explicitly address disrespect and abuse, several women shared comments that reflected negative instances of disrespect and abuse. The use of a tailored framework was deemed necessary to accurately report the prevalence of such experiences among the women who responded to the open-ended question. This highlights the importance of using the ICM RESPECT Toolkit [21] as a framework for analysis.

Table 4 outlines the specific recommendations for improving QMNC provided by participants, which are also discussed in a separate section of the results.

**Fig. 2:**
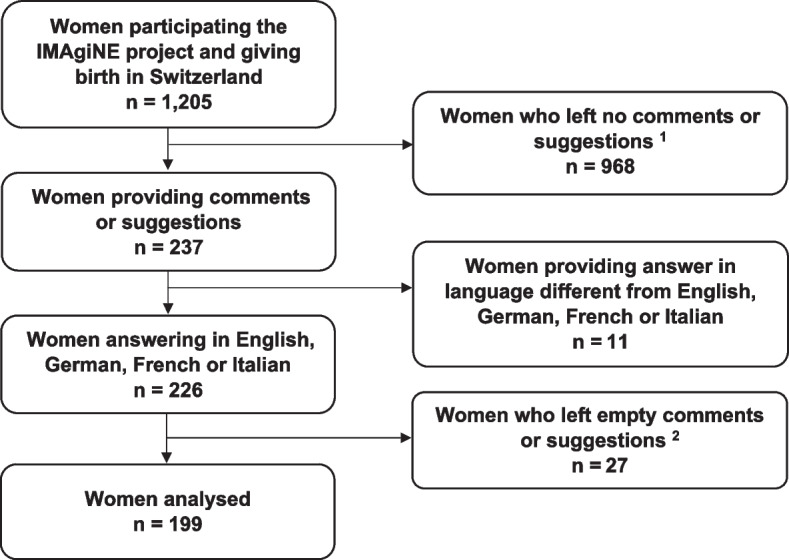
Women participating in the IMAgiNE EURO study and answering to the open-ended question in French, German, Italian or English. *Legend*: ^1^ Women who did not answer to the question “Do you have any suggestions to improve the quality of care in the facility where you gave birth or to improve the questionnaire?”; ^2^ Women who answered by simply indicate: “-”; “no”; “I have no comments”

The qualitative analysis for this study was limited to women who responded to the open-ended question in one of the languages known to the authors (French, German, Italian, English) (Fig. 2). Table 1 summarises the socio-demographic data collected for these women.

**Table 1:** Characteristics of women who gave birth in a Swiss facility and answered the open question in French, German, Italian or English

	***N*** ** = 199**
	n (%)
** Year of birth**	
2020	109 (54.8)
2021	87 (43.7)
2022	3 (1.5)
** Language in which the woman answered the questionnaire**	
French	110 (55.3)
German	55 (27.6)
Italian	23 (11.6)
English	11 (5.5)
** Mother born in Switzerland**	
Yes	135 (67.8)
No	64 (32.2)
** Educational level ** ^**a**^	
Elementary school	0
Junior High school	11 (5.5)
High School	46 (23.1)
University degree	62 (31.2)
Postgraduate degree / Master / Doctorate or higher	80 (40.2)
** Age**	
18–24	2 (1.0)
25–30	42 (21.1)
31–35	89 (44.7)
36–39	46 (23.1)
≥ 40	20 (10.1)
** Parity**	
Primiparous	100 (50.3)
** Birth mode**	
Vaginal spontaneous	110 (55.3)
Instrumental vaginal birth	29 (14.6)
Caesarean section (CS)	
Emergency CS during labour	23 (11.6)
Emergency CS before going into labour	11 (5.5)
Planned or elective CS before going into labour	26 (13.1)
** After birth**	
My baby was admitted to NICU or SCBU	18 (9.0)
I was admitted to ICU	2 (1.0)
** Type of hospital**	
Public	160 (80.4)
Private / clinics	39 (19.6)
** Type of healthcare providers who directly assisted birth**	
Midwife or nurse	185 (93.0)
A student (i.e., before graduation)	44 (22.1)
Obstetrics registrar / medical resident (under post-graduation training)	49 (24.6)
Obstetrics and gynaecology doctor	154 (77.4)
I don’t know (healthcare providers did not introduce themselves)	23 (11.6)
Other	25 (12.6)

*Abbreviations*: *CS* Caesarean Section, *NICU* Neonatal Intensive Care Unit, *SCBU* Special Care Baby Unit, *ICU* Intensive Care Unit, *QMNC* Quality of Maternal and Neonatal Care

^a^Wording on education levels agreed among project partners during the Delphi; questionnaire translated and back translated according to ISPOR Task Force for Translation and Cultural Adaptation Principles of Good Practice. Categories of educational level are not fully aligned with those used in Swiss’ national reports (i.e., compulsory education, secondary level, tertiary level) [30]. Analysis and interpretation considering the educational level as a possible explicative variable should be done with caution

### Data analysis

Women’s comments on the open-ended question were exported to an Excel spreadsheet and then imported into the MAXQDA-20 software, regardless of the language used. This software is suitable for systematically processing the excerpts related to the different themes. Women who answered ‘no’, ‘yes’ or ‘I have no comments’ were excluded from further analysis. Initially, the first author independently coded participants’ comments using MAXQDA-20, along with reflective and analytical notes explaining the decision-making process and choices. The themes sought to summarise women’s experiences of disrespect and abuse in maternity care. For this purpose, women’s comments were analysed thematically, using the categories of disrespect and abuse reported by the ICM [21] as a framework for analysis (i.e., physical abuse; non-consented care; non-confidential care; non-dignified care; discrimination based on specific attributes; abandonment or denial of care; and detention in facilities), following a deductive approach. When necessary, the categories related to disrespect and abuse were operationalised with reference to the systematic review by Sando et al. [35]. If a woman’s statement pertained to two or more categories established in the ICM’s RESPECT Toolkit, the comment was added to both themes. For each language chosen for analysis (i.e., French, German, Italian, English), a second author (CdL, MGE, or SBG) independently coded the same selection of comments, which were compared and discussed. If needed, a third researcher was consulted to reach a consensus. Where themes in the women’s comments were not related to the ICM’s RESPECT Toolkit [21], a reflexive thematic analysis was undertaken following the steps recommended by Braun and Clarke [36, 37], using an inductive approach. Following the iterative process of coding, conceptual sub-themes and other inductive themes were generated. The final list of codes was agreed on during team meetings with experienced maternal and newborn health researchers and all authors of this paper. As part of this publication, all women’s comments were translated into English. The reliability of the translated comments was ensured by double-checking the translation with at least two authors.

### Ethical aspects

The study protocol was approved by the Institutional Review Board of the IRCCS Burlo Garofolo. As this was a voluntary, anonymous survey of maternal views on QMNC, the Ethics Committee of the Canton of Vaud considered that this study did not fall under the Human Research Act (art. 2) and therefore did not require any further ethical approval in Switzerland (CER-VD, information on July 9th, 2021). Data were stored in Italy. Data transmission and storage were secured by encryption. When accessing the link and before participating, women were informed about the aims and methods of the study, including their right to refuse to participate or to withdraw at any time. Informed consent was obtained before answering the questionnaire, and a full privacy statement was available for download if requested.

## Results

Of the 1′205 women who gave birth in Switzerland and participated in the IMAgiNE study, 199 responded to the non-mandatory open-ended question and met the inclusion criteria (Fig. 2). The comments provided by these 199 women were analysed.

In the IMAgiNE EURO questionnaire, participants were asked if they had experienced limitations in QMNC due to the COVID-19 pandemic. Of the 199 women who responded to the open-ended question, 43.2% reported experiencing limitations in the QMNC received due to COVID-19, either ‘always/nearly always’ or ‘sometimes’.

### Characteristics of the study population

The characteristics of the 199 women who responded to the open-ended question are presented in Table 1. Most women (67.8%) were born in Switzerland and responded in French (55.3%). The majority were aged between 31 and 35 years, and 95.5% had completed at least high school. Half of the participants (50.3%) were primiparous, and the vast majority (80.4%) gave birth in a public hospital.

### Categories of disrespect and abuse identified through deductive thematic analysis using the RESPECT toolkit

Table 2 shows the categories of disrespect and abuse according to the RESPECT toolkit and the sub-categories identified.

**Table 2:** Categories of disrespect and abuse according to the ICM’s RESPECT toolkit and the sub-categories identified

**Non-consented care**	
Failing to respect woman’s choices and preferences	
Prioritizing protocols over women’s individuality	
**Non-dignified care (including verbal abuse)**	
Lack of empathy and emotional support from HCP	
Disrespectful attitude and inappropriate gestures towards women	
Transmitting HCP’s own stress to women	
Emotional pressure from HCP	
**Abandonment/neglect of care**	
Ban the presence of a support person or companion during childbirth	
Separate the women from their newborn after birth	
Causing the woman to feel abandoned or ignored (often in a state of pain)	
Fail to provide quality of care	
**Non-confidential care**	
Compromising privacy due to room layout	
**Physical abuse**	
Aggressive behaviours from HCP (i.e. midwives)	
**Discrimination based on specific attributes**	No comments reported by women
**Detention in facilities**	No comments reported by women

*Abbreviation*: *HCP* Healthcare professionals

Overall, five out of the seven categories of disrespect and abuse as defined by the ICM [21] were observed. None of the comments made by women fell into the categories ‘Detention in facilities’ and ‘Discrimination based on specific attributes’.

#### Non-consented care

Comments related to the category of ‘non-consented care’ highlighted instances where HCP failed to respect women’s choices and preferences.

*“Respect the ‘preferences’ for childbirth […], and if medically not feasible, explain in a humane manner and show more understanding”* (comment in German, age ≥ 40, private hospital/clinic).

Some women also felt that the HCP prioritised protocols over women’s individuality.

*“We are* *all lumped into the same group, on the assumption that each of us will give birth, feel, and experience identical emotions”* (comment in French, age 31–35, private hospital/clinic).

It is also worth noting that 11.6% of women reported not knowing the HCP attending their birth because they did not introduce themselves (Table 1). This also highlights an issue related to non-consented care.

#### Non-dignified care (including verbal abuse)

Women reported experiencing various forms of non-dignified care, including a lack of empathy and emotional support from HCP. These elements are crucial, especially during the COVID-19 pandemic and the associated climate of uncertainty.

*“Be more attentive to the needs of mothers despite COVID-19! Be much more empathetic because emotionally it was horrible, and I still haven’t come to terms with this terrible childbirth experience! I feel like my last childbirth was stolen from me*” (comment in French, age 31–35, private hospital/clinic).

Some women also perceived disrespectful attitudes and inappropriate behaviour on the part of HCP.

*“The anaesthetists should be more respectful and take the time instead of adopting inappropriate attitudes and gestures under the pretext of an operation at 7:20 a.m.”* (comment in French, age 25–30, public hospital).

Some women pointed out that HCP often transmitted their own stress to the women in their care.

“*Sometimes, we are faced with exhausted employees who have no filters or who pass on their ‘stress’ to us. In my opinion, this should change*” (comment in French, age 31–35, public hospital).

Finally, women reported emotional pressure from HCP such as being blamed during labour and being shamed for wanting painkillers during childbirth.

“*Shaming for wanting painkillers during labour should not exist—everyone has different pain tolerance and it’s not like there is a medal for suffering*” (comment in English, age 31–35, public hospital).

#### Abandonment/neglect of care

Participants stated that being denied companionship during childbirth constitutes a significant violation of their right to respectful care.

“*The emotional experience of the mother not knowing whether her partner can be present at the birth. […], this was the greatest and most stressful burden. Having a child is not only the mother’s business and leaving the hospital as a father 24 h after the birth or not being able to be there at all is not appropriate*” (comment in German, age 36–39, public hospital).

Being separated from their newborn after birth was also perceived by women as a significant failure in providing respectful care.

“*I gave birth on the day of the second wave of COVID, and my daughter was in the neonatal unit for over three weeks. My partner and I were denied access to the neonatal unit and we had to fight to get into the hospital. It was a nightmare. […] I am being treated for depression because of this. They did not support us during this difficult time due to COVID*” (comment in French, age 25–30, public hospital).

Some women felt left alone and abandoned during maternity care.

“*When mothers give birth without birthing partners they should not be left alone so much, […]. I was given a drug to speed up labour and then left alone. By the time the midwife came back, the baby was crowning, and I was alone. I had to ring an alarm to get them to come back. I heard the midwife say to the other midwife ‘I shouldn’t have left her’, but no one said anything to me*” (comment in English, age ≥ 40, public hospital).

Other testimonies revealed shortcomings in the care provided, mostly due to the lack of available staff, which can be partly attributed to the pandemic context.

“*I experienced 3 bladder hematomas after my caesarean section and felt a pain I had never felt before. […]. During the pain, I thought I was going to die […]. The postpartum service called a doctor, who was busy with a REAL emergency, and never came. Not even the next day. I understand that staff shortages are a reality and solutions must be found*” (comment in French, age 31–35, private hospital/clinic).

#### Non-confidential care

Only six comments were related to the category of ‘non-confidential care’. The most common complaints were about the lack of privacy due to the layout of the rooms in the maternity wards during the COVID-19 pandemic.

“*The only drawback is with regards to the rooms, the separation curtain between the beds is too small and doesn’t provide enough privacy”* (comment in French, age 25–30, public hospital).

The reconfiguration of maternity care in response to the COVID-19 pandemic may have contributed to this problem.

#### Physical abuse

Finally, a few women wrote comments that fall into the category of physical abuse. Some of them reported aggressive behaviour by HCP, particularly midwives.

*“The post-delivery treatment was very difficult for me. The midwives were aggressive, implying that I wasn’t trying hard enough to walk the day after the operation”* (comment in French, age ≥ 40, public hospital).

It should be noted that the woman’s experience may refer to criticism from the midwives rather than physical aggression. The other two comments in this category were testimonies of forced use of instruments during childbirth and pulling of the cord to remove the placenta, which the woman perceived as a direct cause of her subsequent haemorrhage.

### Categories of disrespect and abuse identified through the inductive reflexive thematic analysis

Table 3 presents further categories of disrespect and abuse identified through the reflexive thematic analysis.

**Table 3:** Categories of disrespect and abuse identified through the reflexive thematic analysis

**Lack of sufficient/adequate resources (number of HCP, number of rooms, etc.)**
**No clear information from HCP regarding COVID-19**
Perceived contradictory information
Lack of information about COVID-19 related protective measures
**Inappropriate use of protective equipment by HCP**
**Positive experience**

*Abbreviation*: *HCP* Healthcare professionals

The inductive thematic analysis of women’s comments allowed for the identification of additional themes, most of which were specifically related to the uniqueness of the pandemic context and structural weaknesses within health facilities.

#### Lack of sufficient/adequate resources

Several women emphasized the lack of sufficient and appropriate resources to ensure respectful maternity care, particularly regarding the number of HCP or other material resources such as an adequate number of rooms.

“*The biggest problem was the limited number of staff. When I gave birth, the midwife who was assisting me worked for 13 h without a break, attending to both me and another woman, as well as the mothers in the other rooms. […], we were told to call the midwives only in emergencies, because there were too few of them*” (comment in Italian, age 25–30, private hospital/clinic).

#### No clear information from HCP regarding COVID-19

Some women perceived unclear, contradictory, or insufficient information about protective measures in relation to COVID-19.

“*The guidelines on the viral load at which a Covid* + *mother can visit her child(ren) were not uniformly regulated between the postpartum ward and the NICU; the postpartum ward wanted to keep me in isolation, while the neonatology unit allowed me to visit. This resulted in me not being able to see my children for 5 days after giving birth”* (comment in German, age 36–39, public hospital)*.*

#### Inappropriate use of protective equipment by HCP

A few women also reported inappropriate use of personal protective equipment by HCP.

“*And despite being a private hospital the midwives had to use a disposable mask for the whole shifts (instead of changing every 4 h as recommended)*” (comment in English, age 31–35, private hospital/clinic).

#### Positive experience

Finally, several women reported very positive experiences with the maternity care they received.

*“My experience of childbirth was very positive. I had a very patient and understanding midwife who gave me all the possible choices that could be made at that moment, but this is not the case for everyone, and it shouldn’t be left to chance*” (comment in Italian, age 25–30, private hospital/clinic).

Positive experiences were mainly attributed to a good relationship with HCP, characterised by empathy, effective communication, and the respect of women’s choices during childbirth. These factors were considered essential by women.

*“Excellent communication and empathy from midwives […]. The birth plan was respected, even by the doctors”* (comment in French, age 31–35, public hospital).

### Suggestions to improve QMNC during the pandemic

Table 4 presents specific suggestions for improving QMNC during the pandemic, as identified by participants.

**Table 4:** Suggestions for improving the Quality of Maternal and Neonatal Care (QMNC) during the COVID-19 pandemic

**Allow partners to stay during labour and birth**
**No restrictions on partner and family after childbirth (including older children)**
**Limit the number of visits from other family members**
**No restrictions on NICU visits for parents**

*Abbreviations*: *NICU* Neonatal Intensive Care Unit

#### Allow partners to stay during labour and birth

As mentioned above, the presence of a partner during labour and birth was perceived as essential for women to have a positive childbirth experience. Thus, even during the pandemic, women are advocating for their partners to be allowed to be present during this crucial time.

“*The partner must be allowed to be present during childbirth. It was terrible that the father could only be there at the end and was only allowed to hold the baby briefly before he left and could not come back*” (comment in German, age 31–35, public hospital).

#### No restrictions on partner and family after childbirth (including older children)

Respondents clearly stated that visits from partners and close family members should not have been denied or restricted.

“*Due to the pandemic, the hospital had very strict restrictions regarding the presence of fathers. After the two hours of postnatal observation, the father had to leave the hospital and could not return until the mother and baby were discharged. These rules were very difficult to deal with (loneliness and exhaustion for the mother who had to manage the first few days alone because the staff was overwhelmed, trauma for both parents, […]). These restrictions must be reviewed urgently as they are INHUMANE!”* (comment in French, age 31–35, public hospital).

#### Limit the number of visits from other family members

Several women expressed satisfaction that visits from family members other than their partner were limited. This allowed the women and newborns to rest, and bond.

“*I appreciated the policy of not allowing relatives to visit the postnatal ward. It gave me a chance to rest and bond with my baby. I recommend that this policy be continued”* (comment in German, age 36–39, public hospital).

#### No restrictions on NICU visits for parents

Finally, when a newborn requires admission to the NICU, parents should not be restricted from visiting their child because of the pandemic, as suggested by women.

“*And what about those mothers who have given birth and must have a health pass to see their baby? What kind of world are we living in? It’s a shame”* (comment in French, age 31–35, public hospital).

## Discussion

This study investigated women’s experiences of disrespect and abuse in maternity care during the COVID-19 pandemic in Switzerland. Using the ICM’s RESPECT Toolkit [21] as a framework for analysis, the findings highlighted shortcomings in Swiss health facilities related to disrespect and abuse in maternal care during the pandemic. Women frequently reported mistreatment related to non-consented care, abandonment, neglect, and non-dignified care. Additionally, they feel that HCP often dismiss their wishes and needs with little empathy. Some of the COVID-19 pandemic-related measures, such as denying companionship during childbirth, caused feelings of isolation and loneliness, and some participants conveyed the inhumanity associated with such practices. In times of crisis, certain rights may be restricted in favour of security, safety, or emergency resource management [8]. However, compromising the principles of quality and respectful care poses a significant risk to women and newborns. Negative experiences during pregnancy and the stress generated by the COVID-19 pandemic can have far-reaching consequences for maternal and newborn health, as well as for mother-infant bonding [17, 38–40].

The positive experiences reported by some women also provide encouraging findings. These results underline the importance for HCP, institutions, and policy-makers to recognise that essential elements of respectful maternity care, such as respect, dignity, empathy, and emotional support during childbirth, should be an integral part of care provision and not treated as optional or superfluous in times of health crisis.

### Disrespect and abuse around the time of childbirth

Women’s comments have drawn attention to several complaints of abuse and disrespect in Swiss’ health facilities during the COVID-19 pandemic. These elements are presented in detail in the following sections.

Firstly, healthcare facilities appear to face difficulties to consider women’s choices and preferences during childbirth, yet these elements have been identified as crucial in achieving a positive childbirth experience [41, 42]. Issues related to **non-consented care** are consistent with the quantitative data collected in the Netherlands by van der Pijl et al. [43] among 12′239 women, where almost 40% of the interviewed women reported a lack of choice during labour and birth (e.g., concerning the position to give birth). More specifically in the context of the pandemic, data from a cross-sectional study in Luxembourg show that 42.9% of women were not asked for their consent before instrumental vaginal birth (IVB) [44]. In the present study, women reported that protocols were too often prioritised over their individual needs, which is contrary to the central tenet of patient-centred care [31] and a crucial aspect of high-quality perinatal care as outlined by the WHO [9, 26]. Similar findings were reported in a mixed methods study conducted in Switzerland, where several women reported that their wishes and needs were easily dismissed by HCP, and that interventions were carried out without addressing their concerns [45]. These outcomes suggest that this phenomenon goes beyond the exclusive context of the pandemic, as it appears to be present in healthcare settings regardless of the pandemic context. Nevertheless, it is plausible that the pandemic, with its constantly changing protocols and pressures on HCP [46], may have exacerbated instances of disrespectful care provision, such as non-consented care.

Secondly, during the COVID-19 pandemic, women reported experiencing abandonment and lack of support around the time of childbirth. This was mainly due to pandemic-related measures that restricted the presence and visitation of close family members. These findings corroborate previous studies indicating that hospital-imposed restrictions on partner presence and visiting hours during the COVID-19 pandemic had a negative impact on the experience of pregnancy, childbirth, and the postpartum period, leading to pronounced feelings of sadness and anxiety among women [47] and their partners [48, 49]. Diamond et al. [50] conducted a quantitative study of the impact of perinatal health policy changes resulting from COVID-19 on post-traumatic stress disorder (PTSD) following childbirth. Based on a sample of 269 women in the United States, they found that higher rates of PTSD were significantly associated with limited length of stay (*p* = 0.001) and having only one support person during labour and childbirth (*p* = 0.003) [50]. When suggesting ways to improve QMNC, many women in this study emphasised the importance of not restricting the presence of birth partners and postnatal visits. Interestingly, women were also positive about limiting visits from other family members and friends, which is consistent with existing literature [9, 51]. Healthcare facilities should prioritise flexibility in visitation policies to accommodate parents’ needs during normal times and health crises [17]. It is unclear whether restricting visiting hours has more advantages than disadvantages, and future studies should address this issue. Another major concern expressed by women is the separation from their newborns after childbirth, and many participants argued against restrictions on newborn visitation in the NICU. A global cross-sectional study among 424 HCP [11] found that over a quarter of suspected COVID-19 cases resulted in the separation of mothers from their newborns at birth. This practice, intended for the safety of the baby, has been criticised by Jolivet et al. [8] and Bergman [52], as it can have long-lasting consequences such as impaired attachment, and postnatal depression that persist for months or even years after childbirth, as reported by participants in our study.

Thirdly, health facilities seem to have difficulties in providing dignified care, as women have reported disrespectful attitudes, inappropriate gestures (e.g. pulling on the cord to remove the placenta, slanderous remarks) and a lack of empathy on the part of health professionals. Experiencing such attitudes and emotional pressure from HCP resulted in some women having negative perceptions of their birth experience. Lack of support from HCP has also been found to negatively affect the birth experience in previous research and outside the pandemic context [43]. However, as mentioned by the authors, this type of mistreatment is mostly related to emotional pressure and a lack of empathy, which is more subtle compared to physical abuse or other violent behaviour [43]. Mistreatment of this type seems to be more common in high-income countries (such as Switzerland), where HCP may exhibit abusive and coercive behaviour in a more subtle manner [53]. Nevertheless, it is important to avoid blaming HCP alone, as their behaviour is often influenced by systemic and structural factors [54]. In the pandemic context, a survey among 1′127 health workers from 71 countries [10] found that compromised standards of care, overwhelmed staff coping with rapidly evolving guidelines, and increased infection prevention measures were among the major barriers to providing respectful maternity care.

Fourthly, the issue of compromised privacy in the category of non-confidential care was primarily attributed by women to room layout factors such as the number of patients per room and inadequate space between beds. It is plausible to hypothesise that the pandemic had a negative impact on the provision of confidential care during childbirth in Switzerland, as evidenced by the reconfiguration and closure of maternity wards that occurred amidst the pandemic [2, 55]. This finding should be acknowledged and taken into consideration in future research and maternity care practice.

Finally, a minority of women reported experiencing more serious forms of abuse in health facilities, such as physical abuse. One woman described the midwives as aggressive during the postpartum period. However, based on the general nature of the woman’s comment, her experience may refer to criticism from the midwives rather than physical aggression. Other narratives in this category included accounts of forced use of instruments during childbirth and pulling on the cord to remove the placenta, which the woman perceived as a direct cause of her subsequent haemorrhage. Data from the IMAgiNE EURO study, indicate that 9.6% of women who gave birth during the COVID-19 pandemic in Switzerland experienced some form of abuse, without specifying the type of abuse experienced (physical/verbal/emotional) [27]. This proportion is relatively low compared to other European countries, ranging from 7.0% in Sweden to 23.4% in Serbia [31]. Regardless of whether this finding is directly attributable to the pandemic, such mistreatment should never be tolerated and HCP, institutions and researchers must address these issues to prevent their recurrence in the future, as stated by the WHO [56].

### Lack of resources to cope with the pandemic context

Analysis of women’s comments identified other themes related to disrespect and abuse in maternity care. These themes highlight structural weaknesses in health facilities, such as the critical lack of resources to effectively manage the challenges of the pandemic context while ensuring the provision of respectful maternity care. The lack of human and material resources appears to be perceived by women as directly related to the pandemic context. These qualitative findings corroborate the quantitative results of the IMAgiNE EURO study, which showed that approximately one in five women perceived an insufficient number of HCP as a result of the COVID-19 pandemic [27]. Gaps in emotional support from HCP (mentioned above in the category of non-dignified care) also appear to be partly related to understaffing during the COVID-19 pandemic. Switzerland has the second highest number of physicians and nurses per 1000 inhabitants in the entire European Region, with 4.1 physicians and 17.7 nurses (including midwives) [57]. Despite the relatively high staffing level, the Swiss health care system has been facing long-standing issues even before the pandemic. Notably, there is a persistent shortage of HCP [58] and their working conditions are physically and emotionally demanding [59]. These challenges have impaired the system’s ability to adapt in the face of this unprecedented global health crisis. It is plausible to hypothesise that the pandemic further strained an already fragile system. Some women reported that they were reluctant to seek help from staff, knowing that they were overwhelmed with stress. In some cases, health workers themselves discouraged women from seeking help unless it was considered a real emergency. Health facilities bear the responsibility to create an environment that enables HCP to provide respectful, high-quality maternity care under optimal conditions [25, 60]. Indeed, HCP’ negative attitudes and behaviours are largely dependent on structural factors [54]. The mixed-methods systematic review conducted by Bohren et al. [61] revealed that HCP attributed shortcomings in the healthcare system, such as understaffing, high patient volumes, and long working hours, as contributing to a stressful environment that could lead to unprofessional behaviour. As reported by van der Pijl et al. [42], mistreatment in maternity care can manifest itself in both active and passive behaviours. The former is directly related to the behaviour of the HCP, while the latter is related to the conditions of the health system. This study supports these findings, suggesting that deficiencies in the health care system contribute to disrespect and abuse in maternity care. Improving the attitudes and behaviours of HCP alone will not be sufficient to ensure respectful maternity care. Possible ways of improvement should take into account all stakeholders involved, including professional associations, institutional and political authorities [62].

Although the focus of the present study was on disrespect and abuse in maternity care experienced by women around the time of childbirth during the COVID-19 pandemic, some participants also reported positive experiences of care provided by HCP, such as being treated with empathy, clear communication and respect for their choices. Quantitative results from the IMAgiNE EURO study in Switzerland [27], as well as in other European countries [29–31], show that good care coexists with important QMNC gaps. In line with the salutogenetic approach, it is important to recognise and explore positive experiences around childbirth [63], even during a pandemic. Future research could adopt a salutogenetic framework to explore protective factors, which may be particularly relevant in addressing health crises in the future. In this sense, future research should investigate how respectful maternal care can be maintained. One possible avenue is to use the 10 fundamental rights of childbearing women and newborns outlined in the Respectful Maternity Care (RMC) Charter [20, 64]. Ensuring respectful maternity care is arguably more than the absence of disrespect and abuse. Such investigation could complement the current study and provide valuable insights for health promotion research and practice.

### Strengths and limitations

This study presents original findings in the Swiss context and contributes to a better understanding of disrespectful and abusive practices in facility-based maternity care in high-income countries. To the best of our knowledge, this is the first study in Switzerland to collect women’s experiences around the time of childbirth during the COVID-19 pandemic using the ICM RESPECT toolkit as a framework for analysis. This instrument proved valuable in analysing women’s comments through the lens of disrespect and abuse in maternity care, as many of the participant’s comments fell into the seven categories presented in the toolkit. It was also appropriate to explore women’s experiences during the COVID-19 pandemic. Finally, it is worth noting that the ongoing IMAgiNE EURO project allows for data monitoring. Further research will explore indicators beyond the pandemic.

The limitations of the IMAgiNE EURO study have been described previously [31]. The results of the present study should be interpreted considering the following limitations. First, comments made in an open-ended question on a survey cannot fully replace in-depth interviews with women who have experienced disrespect and abuse around the time of childbirth. Furthermore, the wording of the question “Suggestions for improving the quality of care at the facility level” and its placement at the end of a questionnaire may have influenced participants’ responses, as they had already expressed their opinions about the quality of care. Second, the choice of languages for analysis (German, Italian, French and English) may have excluded the experiences and perspectives of the most vulnerable populations, such as migrant women. As these populations have been particularly affected by the COVID-19 pandemic [27, 65], their perspectives on disrespect and abuse in maternity care should be considered in future research. In addition, their experiences may also vary according to different characteristics (such as language barriers, ethnicity, education, etc.). Third, a possible selection bias cannot be excluded, as it is possible that women who chose to participate were more inclined or interested in the topic. In addition, the use of a non-random, convenience sampling strategy and the non-mandatory nature of the open-ended question may also have introduced the possibility of selection bias (see Fig. 2). It is worth noting that almost all women who responded to the open-ended question had at least a high school education. Thus, the present study may not fully capture the experiences of women with lower levels of education, who may experience different forms of mistreatment or disrespect in health care settings. Forth, as the RESPECT toolkit is designed to support and train HCP to avoid disrespectful and abusive behaviours, it lacks the integration of structural components to assess these practices. These critical aspects were reported by women as important factors contributing to certain disrespectful practices. Future studies investigating disrespect and abuse in maternal care should consider these elements as they shed light on HCP behaviours and the care they provide. Finally, while the results indicate that disrespect and abuse during labour and childbirth do occur in Switzerland, the qualitative design of the study does not provide insight into the prevalence of these experiences. Very few studies [43] have examined the prevalence of disrespect and abuse in facility-based maternity care in high-income countries according to the seven categories developed by Bowser et al. [19]. Further research is needed to thoroughly investigate the occurrence of disrespectful and abusive maternity care in Swiss facilities.

## Conclusion

The collection of women’s experiences during the COVID-19 pandemic in Switzerland proved to be highly relevant in identifying disrespectful and abusive practices in maternal care. This study highlights the importance of studying these aspects, even in high-income countries, as the results reveal certain inappropriate care practices that call for action to ensure respectful maternity care in Switzerland. The pandemic has undoubtedly played a role in compromising certain aspects of respectful care, such as patient choice [66] and dignified care. However, the COVID-19 pandemic has also acted as a magnifying glass, revealing and exacerbating pre-existing gaps in respectful maternity care [24] and structural weaknesses in health facilities. Not only should HCP pay attention to women’s distress signs and empathetically meet their needs to avoid disrespect and abuse in maternity care, but they should also have the necessary resources to do so. These lessons need to be translated into advocacy, as policy-makers and health facilities should ensure that HCP have sufficient resources to provide quality and respectful maternity care, even in times of health crisis.

### Supplementary Information

Supplementary Material 1.

## Data Availability

The datasets used and/or analysed during the current study are available from the corresponding author on reasonable request.
