# Supplementary material for: Women’s experiences of disrespect and abuse in Swiss facilities during the COVID-19 pandemic: a qualitative analysis of an open-ended question in the IMAgiNE EURO study
Source: BMC Pregnancy Childbirth. 2024 May 31;24:402. doi: 10.1186/s12884-024-06598-6 (PMC11143635; doi:10.1186/s12884-024-06598-6)
Supplement: Supplementary file 1 — Supplementary Material 1. [file 12884_2024_6598_MOESM1_ESM.docx]

# Supplementary files

**Supplementary Information 1**: Standards for Reporting Qualitative Research (SRQR); <https://www.equator-network.org/reporting-guidelines/srqr/>.

|  |  | **Page/line no(s).** |
| --- | --- | --- |
| **Title and abstract** | |  |
|  | **Title** - Concise description of the nature and topic of the study Identifying the study as qualitative or indicating the approach (e.g., ethnography, grounded theory) or data collection methods (e.g., interview, focus group) is recommended | Title, lines 2-6 |
|  | **Abstract** - Summary of key elements of the study using the abstract format of the intended publication; typically includes background, purpose, methods, results, and conclusions | Abstract section, lines 9-29 |
| **Introduction** | |  |
|  | **Problem formulation** - Description and significance of the problem/phenomenon studied; review of relevant theory and empirical work; problem statement | Background section of the manuscript, lines 35-129 |
|  | **Purpose or research question** - Purpose of the study and specific objectives or questions | Background section of the manuscript, lines 130-137 |
| **Methods** | |  |
|  | **Qualitative approach and research paradigm** - Qualitative approach (e.g., ethnography, grounded theory, case study, phenomenology, narrative research) and guiding theory if appropriate; identifying the research paradigm (e.g., postpositivist, constructivist/ interpretivist) is also recommended; rationale** | Data analysis section of the manuscript, lines 208-233 |
|  | **Researcher characteristics and reflexivity** - Researchers’ characteristics that may influence the research, including personal attributes, qualifications/experience, relationship with participants, assumptions, and/or presuppositions; potential or actual interaction between researchers’ characteristics and the research questions, approach, methods, results, and/or transferability | Ethical aspects section of the manuscript, lines 206-208 and lines 234-236 |
|  | **Context** - Setting/site and salient contextual factors; rationale** | Study design and Data collection sections of the manuscript, lines 140-148 and lines 153-188 |
|  | **Sampling strategy** - How and why research participants, documents, or events were selected; criteria for deciding when no further sampling was necessary (e.g., sampling saturation); rationale** | Data collection section of the manuscript, lines 153-188 |
|  | **Ethical issues pertaining to human subjects** - Documentation of approval by an appropriate ethics review board and participant consent, or explanation for lack thereof; other confidentiality and data security issues | Ethical aspects section of the manuscript, lines 242-256 |
|  | **Data collection methods** - Types of data collected; details of data collection procedures including (as appropriate) start and stop dates of data collection and analysis, iterative process, triangulation of sources/methods, and modification of procedures in response to evolving study findings; rationale** | Data collection section of the manuscript, lines 153-188 |
|  | **Data collection instruments and technologies** - Description of instruments (e.g., interview guides, questionnaires) and devices (e.g., audio recorders) used for data collection; if/how the instrument(s) changed over the course of the study | Data collection section of the manuscript, lines 153-164 |
|  | **Units of study** - Number and relevant characteristics of participants, documents, or events included in the study; level of participation (could be reported in results) | Data collection and Results sections of the manuscript, lines 169-172, lines 258-262, and Table 1 |
|  | **Data processing** - Methods for processing data prior to and during analysis, including transcription, data entry, data management and security, verification of data integrity, data coding, and anonymization/de-identification of excerpts | Data analysis section of the manuscript, lines 198-240 |
|  | **Data analysis** - Process by which inferences, themes, etc., were identified and developed, including the researchers involved in data analysis; usually references a specific paradigm or approach; rationale** | Data analysis section of the manuscript, lines 198-240 |
|  | **Techniques to enhance trustworthiness** - Techniques to enhance trustworthiness and credibility of data analysis (e.g., member checking, audit trail, triangulation); rationale** | Data analysis section of the manuscript, 234-240 |
| **Results/findings** | |  |
|  | **Synthesis and interpretation** - Main findings (e.g., interpretations, inferences, and themes); might include development of a theory or model, or integration with prior research or theory | Results section of the manuscript, lines 258-520, and Tables 2-4 |
|  | **Links to empirical data** - Evidence (e.g., quotes, field notes, text excerpts, photographs) to substantiate analytic findings | Results section of the manuscript, lines 269-520 |
| **Discussion** | |  |
|  | **Integration with prior work, implications, transferability, and contribution(s) to the field -** Short summary of main findings; explanation of how findings and conclusions connect to, support, elaborate on, or challenge conclusions of earlier scholarship; discussion of scope of application/generalizability; identification of unique contribution(s) to scholarship in a discipline or field | Discussion section of the manuscript, lines 521-766 |
|  | **Limitations** - Trustworthiness and limitations of findings | Strengths and limitations section of the manuscript, lines 767-834 |
| **Other** | |  |
|  | **Conflicts of interest** - Potential sources of influence or perceived influence on study conduct and conclusions; how these were managed | Cover letter, Declarations section of the manuscript |
|  | **Funding** - Sources of funding and other support; role of funders in data collection, interpretation, and reporting | Declarations section of the manuscript |
|  | *The authors created the SRQR by searching the literature to identify guidelines, reporting standards, and critical appraisal criteria for qualitative research; reviewing the reference lists of retrieved sources; and contacting experts to gain feedback. The SRQR aims to improve the transparency of all aspects of qualitative research by providing clear standards for reporting qualitative research. | |
|  | **The rationale should briefly discuss the justification for choosing that theory, approach, method, or technique rather than other options available, the assumptions and limitations implicit in those choices, and how those choices influence study conclusions and transferability. As appropriate, the rationale for several items might be discussed together. | |

**Reference:**

O'Brien BC, Harris IB, Beckman TJ, Reed DA, Cook DA. **Standards for reporting qualitative research: a synthesis of recommendations.** *Academic Medicine*, Vol. 89, No. 9 / Sept 2014. DOI: 10.1097/ACM.0000000000000388
